# Supplementary material for: Association between oral microbiome and seven types of cancers in East Asian population: a two-sample Mendelian randomization analysis
Source: Front Mol Biosci. 2023 Nov 21;10:1327893. doi: 10.3389/fmolb.2023.1327893 (PMC10702768; doi:10.3389/fmolb.2023.1327893)
Supplement: Supplementary file 1 [file DataSheet1.ZIP › Supplementary Materials/MR plots for tongue/tongue═╝/Pancreatic cancer/╥╚╧┘░⌐_╔╕╤í╜ß╣√/pheno.2323_to_pancreatic cancer_leave_one_out.pdf]

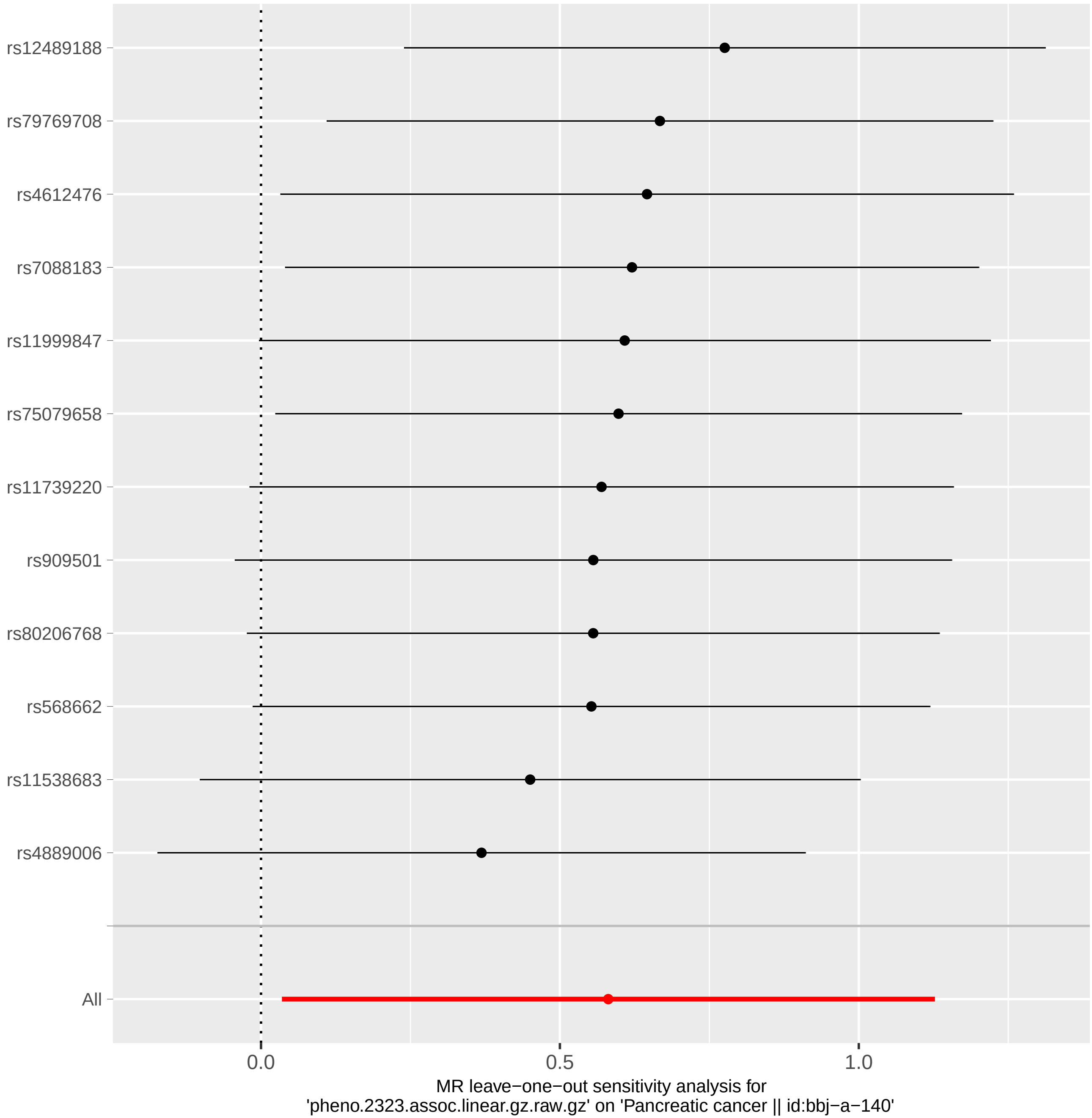

rs12489188
rs79769708
rs4612476
rs7088183
rs11999847
rs75079658
rs11739220
rs909501
rs80206768
rs568662
rs11538683
rs4889006
All
0.0
0.5
1.0
MR leave−one−out sensitivity analysis for
'pheno.2323.assoc.linear.gz.raw.gz' on 'Pancreatic cancer || id:bbj−a−140'
